# Role of Frizzled receptor expression on patients’ survival with gastrointestinal cancers: A systematic review with meta-analysis

**DOI:** 10.22088/cjim.13.1.1

**Published:** 2022

**Authors:** Nasim Hafezi, Reza Alizadeh-Navaei, Monireh Golpour, Parisa Zafari, Abolghasem Ajami

**Affiliations:** 1 Department of Immunology, School of Medicine, Mazandaran University of Medical Sciences, Sari, Iran; 2 Molecular and Cell Biology Research Center, Student Research Committee, Mazandaran University of Medical Sciences, Sari, Iran; 3 Gastrointestinal Cancer Research Center, Non-Communicable Diseases Institute, Mazandaran University of Medical Sciences, Sari, Iran; 4 Department of Infectious Diseases, Antimicrobial Resistance Research Center, Mazandaran University of Medical Sciences, Sari, Iran

**Keywords:** Frizzled receptor, Expression gastrointestinal cancer, Survival

## Abstract

**Background:**

Frizzled receptors (FZD) play a pivotal role in the initiation and progression of a wide array of cancers. Dysregulated expression of FZD receptors is correlated with higher metastasis and invasive potential, as well as short survival in many malignancies. In this meta-analysis, we aimed to verify the prognostic value of FZD receptor expression on patients’ survival with different types of gastrointestinal (GI) cancers, including gastric, colorectal, and esophageal cancers.

**Methods:**

A systematic search was performed using PubMed, Scopus, and Web of Science from 2000 to November 2020. Fourteen studies, including 2997 patients met our inclusion criteria, in which nine articles were considered FZD7 while the rest were about other FZD members. The fixed-effect model was used to estimate the pooled hazard ratio (HR) and the 5-year overall survival (OS) rate. We used the Newcastle-Ottawa scale of cohort articles to determine the quality of included studies.

**Results:**

The results showed that high expression of FZD receptors is associated with the poor survival in patients with GI cancers (HR= 1.83, 95% CI: 1.5–2.17). Moreover, multivariate analysis indicated that FZD receptors could be considered as an independent prognostic factor (HR = 1.76, 95% CI: 1.37–2.16).

**Conclusion:**

According to our results, overexpression of FZD receptors predicts a poor prognosis in patients with GI cancers and could be used as a useful therapeutic target.

Gastrointestinal (GI) cancers including gastric (GC), colorectal (CRC), and esophageal (EC) cancers are the major causes of cancer-related death in the world. Early diagnosis of cancers shows a remarkable overall survival (OS) compared with advanced stages. In GI cancers, the 5- year OS decreased from 43-90% in the early stages to 4-14% in the end-stages (1). Identifying a novel molecule predicting the outcome of GI cancers could lead to a timely and appropriate treatment. The Wnt signaling pathway is one of the most important dysregulated pathways involved in tumor growth and progression. It is composed of two separate pathways named canonical and noncanonical. Disruption of any component of this pathway is associated with the onset and progression of cancer through the formation of cancerous stem cells, abnormal cell proliferation, and invasion. FZD receptors (FZDs) are seven-transmembrane proteins that consist of ten members (FZD 1-10) with high sequence homology, which is composed of a conserved extracellular cysteine-rich domain (CRD), a conserved seven-pass transmembrane region, and a cytoplasmic domain (2).

Activation of Wnt signaling happens following the interaction between Wnt ligands and CRD domain of FZDs. Consistent with available evidence, the interaction between Wnt and FZDs is irregular, and each of the Wnt family member can bind to more than one FZD receptor (2). Overexpression of FZD family members has been reported in several cancers and it is related to invasive and metastasis potential of tumor (3). 

For example, in GI cancers, the high expression of FZDs has been demonstrated to have major functions during the tumor formation and progression (4-6). Accordingly, targeting FZDs with monoclonal antibodies or peptide inhibitors is currently being investigated as a new cancer therapy approach. (7-10). 

According to the importance of dysregulated FZDs in the development and exacerbation of cancers and also having a potential to a possible therapeutic target, no systematic research assessed the reliability of FZDs as an indicator of prognosis in patients with GI cancer. So, this meta-analysis was conducted to elucidate the association between FZDs expression and the survival of digestive system cancers including GC, CRC, and EC. 

## Methods

**Search strategy: **This meta-analysis evaluated the association of high and low levels of FZDs expression with the survival of patients with GI cancers. The search was conducted using the database of Scopus, Web of Science, and PubMed from 2000 to November 2020 in the title and abstract by the following keywords: 

(FZD1 OR FZD-1 OR "frizzled class receptor 1" OR "frizzled class receptor -1" OR FzE1 OR FzE-1 OR frizzled1 OR frizzled-1 OR fz1 OR fz-1 OR hFz1 OR hFz-1 OR "frizzled family receptor 1" OR "frizzled family receptor-1" OR "Frizzled Homolog 1" OR "Frizzled Homolog-1"…… Frizzled Homolog-10) and (cancer OR neoplasm OR tumor OR malignancy OR carcinoma OR cancers OR neoplasms OR tumors OR malignancies OR carcinomas OR carcinogenesis OR oncogeneses OR tumorigenesis OR tumorigeneses OR oncogenesis OR oncogeneses) and (survival OR "overall survival" OR OS OR "disease specific survival" OR DSS OR "disease free survival" OR DFS OR "survival rate" OR "poor survival" OR "survival analysis" OR " progression free survival" OR " OR PFS OR "Kaplan Meier estimate" OR "cumulative survival" OR "CUM survival" OR "treatment free survival" OR TFS OR "survival curve" OR "survival curves" OR "Kaplan-Meier curve" OR "Kaplan-Meier curves" OR "event free survival" OR EFS). 

Screening of documents was conducted in three steps. First, removing duplicated articles. Second, selecting relevant articles based on title and abstract. Third, reviewing the details of the full text of all selected articles. Finally, the references of all included articles were reviewed to identify further studies. 

**Inclusion and exclusion criteria: **Studies with the following inclusion criteria were included: 1) studies in human populations with gastrointestinal cancers including colorectal, gastric, and esophageal cancers 2) studies that have evaluated the survival of patients based on dysregulated FZD receptor expression, 3) studies that have analyzed the Kaplan Meier curve or reported the survival rate or hazard ratio (HR). 

The exclusion criteria were: (1) non-original publications including systematic reviews, meta-analyses, reviews, commentaries, conference abstracts, and letters; (2) studies about the other prognosis markers and cancers (3) duplicative publications; (4) non-English-language and (5) animal studies. Two authors independently searched databases, and any discrepancies were resolved by consultation with the supervisor.

**Data extraction and quality assessment: **The following information was collected from each study: first author name, year, country, type of FZD receptor, tumor type, sample size, detection technique, follow-up time, survival rate, and hazard ratio (HR) of low and high FZDs expression for OS with 95% confidence intervals (CI). Evaluation of the quality of all selected studies was measured according to the Newcastle‐Ottawa Quality Score (NOS) for cohort studies. Studies were scored based on the three parameters: selection, comparability, and exposure by the star scoring method with a range of lowest quality (0 stars) to the highest quality (13 stars). A score of ≥6 indicated good quality.

**Statistical analysis: **HR or 5-year OS with 95% CI was used to evaluate the association between the high and low levels of FZD receptors and OS. In articles that did not mention the survival rate, the rate of 5-year OS was manually measured from survival plots. Statistical heterogeneity was examined with *I*^2^ statistics. 

With *I*^2^ ˂ 50%, pooled data were conducted with a fixed-effect model. Publication bias was determined by utilizing Egger’s test. Data were analyzed by Stata statistical software Version 11.

**Figure 1:**
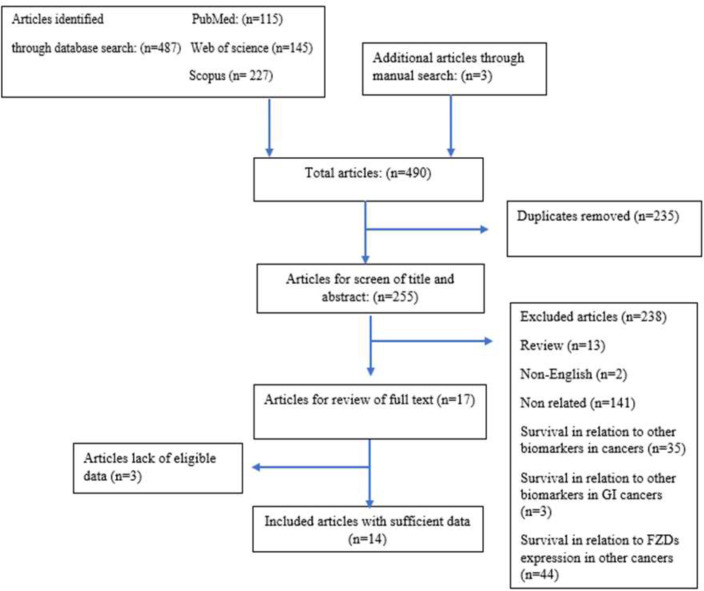
Flow chart of the selection process of eligible studies

**Abbreviation**: n: number of articles, OS: overall survival, HR: hazard ratio

## Results

**Literature research: **A total of 487 studies between 2000-2020 were identified from the database search. After removing duplicate studies, 255 articles were reviewed for title and abstract screening. Subsequently, 238 articles were excluded in this step as they did not have the eligible inclusion criteria.

 Then, 17 potentially relevant papers were selected for full-text assessment, and three articles were excluded because of the lack of data to estimate the survival rate. Finally, 14 studies were included in this meta‐analysis. The screening process of candidate studies is presented in details in figure 1. 

**Patients’ characteristics: **In the pooled analysis of 14 studies, a total of 2997 patients, including 1503 patients with high expression of FZD receptors and 1494 patients with low expression of FZD receptors were included in this meta-analysis.

 In nine studies, survival was analyzed in relation to FZD7 expression and other studies were about FZD2, FZD3, FZD4, FZD6, and FZD8, one study for each member. All of the samples were tissue biopsies of primary tumors. Among the articles, eleven studies were performed in China, two in Germany, and one in Japan. The main information of the eligible studies was shown in table 1.

**Table 1:** Characteristics of the included studies

**Author/year**	**Type of FZD**	**Type of cancer**	**Method**	**Cut off** **of high FZD**	**No of patients with high FZS/ low FZD EXP**	**5 -year OS rate high FZD**	**5 -year OS rate low FZD**	**U HR (95% CI)**	**M HR (95% CI)**	**Follow- up (month)**	**Quality score**
Cao et al., 2017(11)	Fzd7	ESCC	Microarray-IHC	>192.5^a^	165/87	0.06	0.11	1.708 (1.234-2.363)	1.709 (1.235-2.365)	Max 63.33	9
Li et al., 2018(12)	Fzd7	GC	IHC	IRS>4	100/151	0.30	0.65	2.489 (1.767-3.507)	1.681 (1.153-2.45)	Max 150 Mean 57.1	9
Schmuck et al., 2011(13)	Fzd7	GC	Microarray-IHC	IRS≥1	131/229	0.03*	0.1*	NA	NA	Max 127.2 MED 19	7
Freiin Grote et al., 2019(14)	Fzd7	GC	IHC	IRS≥4	44/52	0.31*	0.28*	NA	NA	Max 120	7
Luo et al., 2019(15)	Fzd7	GC	RNA- seq	NA	204/204	0.21*	0.45*	1.56 (1.1-2.22)	NA	Max 125	6
Cheng et al., 2019(16)	Fzd7	GC	RNA-microarray	NA	301/330	0.4*	0.52*	NA	NA	Max 152.4	6
Pi et al.,2020(17)	Fzd7	GC	IHC	NA	40/39	0.25*	0.4*	NA	NA	Max 110.8	7
Ye et al., 2019(18)	Fzd7	CRC	IHC	NA	68/46	0.25*	0.82*	2.459 (1.177-5.263)	1.606 (0.73-3.532)	Max 106	9
Ueno et al., 2009(19)	Fzd7	CRC	Real-time PCR	c	12/109	0.62*	0.86*	NA	NA	Max 105	7
Fu et al., 2020(20)	Fzd2	EAC	IHC	IRS≥2	69/31	0.14*	0.31*	NA	NA	Max 98	7
Li et al., 2019(21)	Fzd4	GC	NA	NA	292/100	0.25*	0.41*	NA	NA	Max 125	7
Zhang et al., 2020(22)	Fzd6	ESCC	IHC	d	15/80	0.14	0.37	2.053 (1.221-3.45)	2.541 (1.482-4.356)	MED 30 Max 93.5	8
Xu et al., 2016(23)	Fzd8	CRC	IHC	IRS≥4	35/28	0.54*	0.85*	4.46 (1.51-13.19)	3.55 (1.17-10.79)	Max 92	8
Wong et al., 2013(24)	Fzd3	CRC	ICC	>327 ^b^	27/8	0.34*	0.78*	NA	NA	Max 42	6

**Abbreviations**: CRC: colorectal cancer, ESCC: esophageal squamous cell carcinoma, GC: gastric cancer, HR: hazard ratio, IHC: Immunohistochemistry, ICC: Immunocytochemistry, M: multivariate, Max: maximum, MED: median, NA: not available, OS: overall survival, U: univariate, a: IHC score, b: ICC score, c: >mean of all case, d: score of 2 + and 3 +, * estimate manually from Kaplan Meier curve

**Fig 2:**
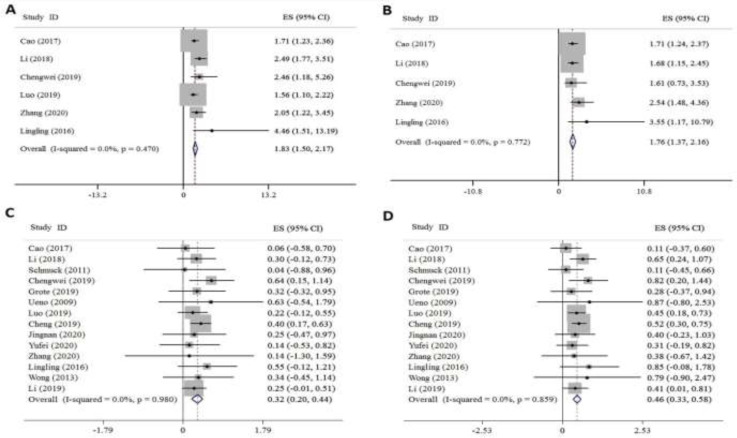
Forest plot of OS in all GI cancer patients is associated with: A. univariate HR for OS, B. multivariate HR for OS, C. high FZD expression, D. low FZD expression

Abbreviations: FZD, Frizzled; GI, gastrointestinal

**Impact of FZD receptors expression on OS in all GI patients: **The rate of survival has been shown as HR in 6 out 14 included articles. But the 5-year OS rate was available in all studies. No heterogeneity was observed among studies, and the data was analyzed using the fixed-effect model. From the combined survival data of patients, the following results were obtained. Analysis of the pooled HR showed a positive correlation between high FZDs expression and poor OS (HR= 1.83, 95% CI: 1.5–2.17, Fig. 2A). Egger’s test indicated no publication bias (P=0.197). Other interfering factors including sex, age, tumor differentiation, lymph node metastasis, and stage were reported in 5 studies. The multivariate analysis indicated that high expression of FZDs is an independent prognostic factor for cancer survival (HR = 1.76, 95% CI: 1.37–2.16, fig. 2B). In addition, there was no publication bias based on Egger’s test (P=0.241). Five-year survival rate comparison between the two groups of patients with high and low level of FZDs showed that overexpression of FZDs in patients with GI cancer was associated with worse OS compared with those of low expression (0.32, CI 95%: 0.2- 0.44 versus 0.46, CI 95%: 0.33- 0.58, fig. 2C, D). Also, there was no publication bias for data of high expression FZD group based on Egger’s test (P=0.759) and low expression one (P=0.962).

**Fig 3:**
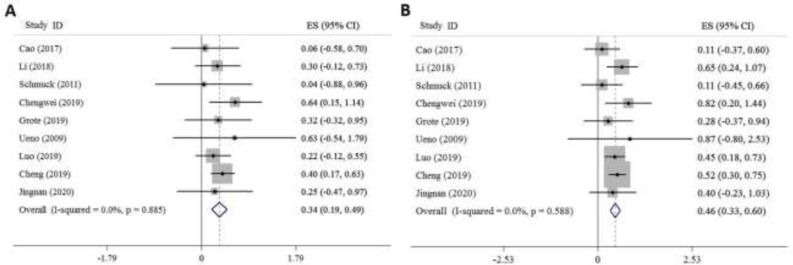
Forest plot of OS in all GI cancer patients is associated with: A. high FZD7 expression, B. low FZD7 expression

**Sensitivity analysis: **Sensitivity analysis showed that the 5-year OS rate in GI patients with a high level of FZD7 expression was associated with poor survival than those with the low level of FZD7 expression (0.34, 95% CI: 0.19-0.49 versus 0.46, 95% CI: 0.33-0.6, fig. 3A, B) 

**Fig 4:**
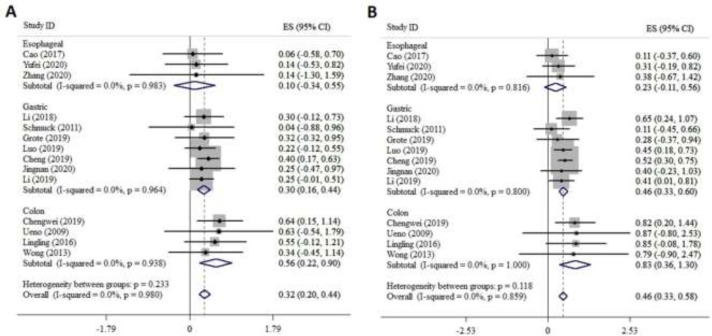
Forest plot of OS in EC, GC, and CRC patients is associated with: A. high FZD expression, B. low FZD expression

Abbreviations: EC, esophageal cancer; GC, gastric cancer; CRC, colorectal cancer

**Subgroup analysis: **Subgroup analysis was performed based on cancer type to examine the possible association between FZDs expression and OS in EC, GC, and CRC patients. The pooled analysis of three studies on EC showed that the 5-year OS rate was related to poor survival in patients with high level of FZDs expression than those with low level (0.1, 95%CI: -0.34-0.55 versus 0.23, 95%CI: -0.11-0.56, fig. 4A, B). The pooled analysis of seven studies on GC showed that patients with a high level of FZDs have lower 5-year OS than those with low levels (0.3, 95% CI: 0.16-0.44 versus 0.46. 95% CI: 0.33-0.6, fig. 4A, B). The pooled analysis of four studies on CRC showed the 5-year OS was lower in patients with a high level of FZDs expression than those with a low level (0.56, 95% CI: 0.22-0.9 versus 0.83, 95% CI: 0.36-1.3, fig. 4A, B). 

## Discussion

**Fig 5:**
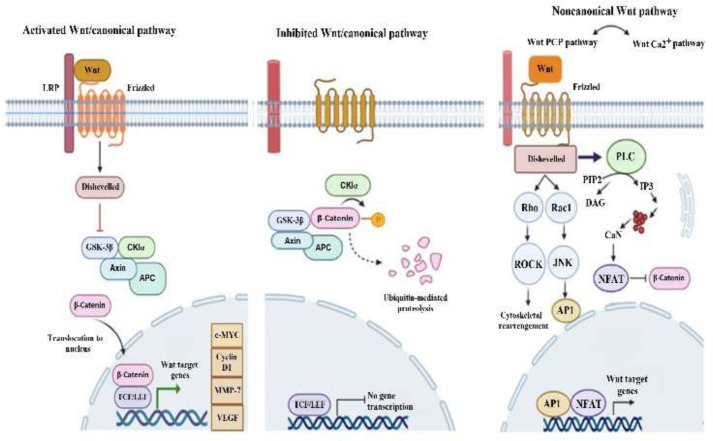
A. In canonical pathway, binding of a Wnt ligand to a frizzled receptor triggers a signaling cascade contributes to beta-catenin translocation to the nucleus. Beta-catenin binds to the T-cell factor/lymphoid enhancer factors (TCF/LEFs) transcription factors mediating the upregulation of Wnt target genes. In the absence of Wnt ligands, cytoplasmic beta-catenin molecules bind to a destruction complex which phosphorylates and targets β-catenin for proteasomal degradation. B. In non-canonical Wnt/planar cell polarity (PCP) pathway, the binding of Wnt ligands to frizzled receptors activates Rho and Rac small GTPases leading to the cytoskeletal changes and gene transcription. In Wnt/Ca2+ pathway, PLC (phospholipase C) activation leading to the release of intracellular Ca++, then activates Ca++ dependent signaling pathways like CaN (calcineurin) /NFAT (nuclear factor of activated T cells) (Figure was constructed using Biorender (https://biorender.com))

This study meta-analyzed the survival of patients with GI cancers based on the high and low level of FZDs expression. We first analyzed the overall impact of FZDs expression on survival in all GI cancers. According to the pooled HR analysis which indicates the relative risk of death in patients with high level of FZDs than those with low level, FZDs overexpression leads to a 1.83-fold increase in the risk of death. Similarly, a comparison of a 5-year OS between patients with high and low of FZDs expression showed a lower survival rate in patients with high FZD expression. Moreover, the results of subgroup analysis based on GI cancer types revealed that the higher level of FZDs expression is associated with lower survival. Therefore, the high level of FZDs may be a predictive value for prognosis of GI cancers. According to included studies, FZD7 is the most frequently studied member with prognostic importance among the 10 FZD family members. Also, the survival rate in patients with a high level of FZD7 was correlated with an increase in the odds of death than those with a low level. In recent years, FZDs have been identified as tumor-associated antigens. The protein level of FZDs is elevated in a wide range of cancers including both solid tumors and hematologic malignancies (25). Up-regulation of the FZDs contributes to tumorigenesis by affecting different cellular processes, including proliferation, invasion, metastasis, angiogenesis, stemness, drug resistance, and survival (26-29). Following the present finding, a meta-analysis from the Oncomine database revealed a negative correlation between the FZD7 expression with the event and relapse-free survival in CRC patients (18). On the contrary, FZDs overexpression may refer to a favorable prognosis. Yan et al. observed an elevated level of FZD6 which is associated with decreased GC cell proliferation and migration by activation of the noncanonical Wnt pathway. (30). In our included studies, Antonia Freiin Grote et al. observed that GC patients with high FZD7 expression have a higher survival rate than those with low FZD7 expression, but it was not statistically significant (14). A positive correlation between the expression of FZDs and cancer survival has also been reported in other types of cancer. For example, in patients with lung cancer, higher FZD2 expression was associated with improved survival; also, in patients with high-grade serous carcinoma after chemotherapy, the high level of FZD1 was associated with longer OS (31, 32). Prognostic consequences of FZDs overexpression arises from distinct downstream pathways. Most of the FZDs are involved in both canonical and noncanonical Wnt signaling pathways. There is a consensus on the canonical Wnt pathway in driving cancers with aggressive features and poor outcomes. In the canonical pathway, the FZDs-Wnt interaction leads to the stimulation of a complex downstream molecules which are required for activation of β-catenin transcription factor and its translocation to the nucleus. This interaction activates target genes like c-myc, cyclin d1, matrix metalloproteinase and vascular endothelial growth factor (VEGF) that are involved in various processes of tumorigenesis, including tumor initiation, tumor growth, cell senescence, cell death, differentiation and metastasis. On the other hand, the noncanonical Wnt pathways (including the wnt/Ca2+, wnt/planar cell polarity) are activated independent of β-catenin and have been implicated in both oncogenic or tumor suppressor functions (33) (Fig. 5A ,B). 

According to the various types of ligands and receptors in this pathway, it seems that the type of ligand and receptor combination as well as the type of tumor affect the noncanonical pathway outcome result in the activation of oncogenic or tumor-suppressive genes.

In our meta-analysis, the following limitations should be considered. The number of studies was limited. Studied patients in included papers were both new-case and under-treatment. A standardized cut-off point was not available for the measurement of the high and low levels of FZDs as well as the lack of more accurate techniques such as flow cytometry than IHC and molecular methods. In most studies, the HR rate has not been reported and we did not analyze HR in other ways. Except of the three studies, the 5-year survival was estimated manually from the Kaplan–Meier curves. In conclusion, our study confirmed that FZDs overexpression is associated with shorter survival and could be considered as a candidate molecule for prognostic assessment and therapeutic targets in human GI cancers. However, due to the above limitations, additional studies are warranted to confirm these results. 

In conclusion, we identified that FZDs expression could be considered as an important regulator in GI cancers. Accordingly, targeting FZDs might be an attractive therapeutic strategy for GI patients.

## Conflict Of Interest:

The authors report no conflicts of interest.

## Prospero Registration:

CRD42020222259
